# Demographic and socioeconomic risk factors for pain progression and recurrence in middle-aged and older adults: multistate analysis of a prospective English cohort study

**DOI:** 10.1093/ageing/afaf364

**Published:** 2026-01-03

**Authors:** Mikaela Bloomberg, Feifei Bu, Daisy Fancourt, Andrew Steptoe

**Affiliations:** University College London Institute of Epidemiology and Health Care - Department of Epidemiology and Public Health, 1-19 Torrington Place, London, WC1E 7HB, United Kingdom of Great Britain and Northern Ireland; University College London Institute of Epidemiology and Health Care - Department of Behavioural Science and Health, 1-19 Torrington Place, London, WC1E 7HB, United Kingdom of Great Britain and Northern Ireland; University College London Institute of Epidemiology and Health Care - Department of Behavioural Science and Health, 1-19 Torrington Place, London, WC1E 7HB, United Kingdom of Great Britain and Northern Ireland; University College London Institute of Epidemiology and Health Care - Department of Behavioural Science and Health, 1-19 Torrington Place, London, WC1E 7HB, United Kingdom of Great Britain and Northern Ireland

**Keywords:** pain, chronic pain, social determinants of health, pain severity, pain trajectory, older people

## Abstract

**Background:**

While demographic and socioeconomic factors such as female sex and socioeconomic disadvantage are well-established risk factors for pain onset, previous studies examining long-term pain trajectories give mixed results and often overlook how pain fluctuates. This study identified demographic and socioeconomic risk factors for pain progression, remission and recurrence using multistate models to capture the dynamic nature of long-term pain.

**Methods:**

Data were drawn from 9369 adults aged 50–98 from the English Longitudinal Study of Ageing (study years: 2002/03–2021/23). The baseline wave for each participant was their first wave of pain (moderate–severe or mild pain). Pain severity at subsequent waves was categorised into three states: (i) moderate–severe; (ii) mild; or (iii) none. We used multistate models to examine associations of demographic and socioeconomic factors with pain improvement (state 1–2), worsening (state 2–1), remission (state 1–3) and recurrence (state 3–1).

**Results:**

Findings particularly highlighted sex and socioeconomic disparities: compared with males, females were less likely to experience pain improvement (hazard ratio [HR] = 0.84, 95% confidence interval = 0.74–0.96) or remission (HR = 0.72, 0.64–0.80), and more likely to experience recurrence (HR = 1.45, 1.25–1.68). More education was associated with pain improvement (HR = 1.43, 1.16–1.76) and remission (HR = 1.30, 1.07–1.58), and lower risk of worsening (HR = 0.52, 0.42–0.64) and recurrence (HR = 0.67, 0.52–0.85); similar patterns were observed for wealth, with greater wealth associated with more favourable pain trajectories.

**Conclusion:**

Pain fluctuates over time, following socially patterned trajectories, with women and socioeconomically disadvantaged individuals more likely to experience persistent or recurring pain. These findings highlight the importance of risk-stratified approaches, including proactive monitoring and management.

## Key Points

- Pain in older adults is highly dynamic, with frequent transitions between worsening, remission and recurrence over time.
- Women and socioeconomically disadvantaged individuals are more likely to experience persistent or recurring pain.
- Findings support the need for equity-informed pain management strategies.

## Introduction

Chronic pain is a major public health concern, contributing to disability, reduced quality of life and substantial healthcare costs [1]. This is a particularly relevant issue for older adults: in the UK, prevalence estimates for chronic pain range from 14% in adults aged 18–25 years, to over 60% in adults aged 75 years and above [2]. Other demographic and socioeconomic characteristics such as being female and less education are also well-established risk factors for incident pain [3] and impact quality and accessibility of pain therapies [4]. However, many pain conditions are intermittent or progressive, characterised by recurrent or worsening pain [5] and even pain that does not meet the definition for chronicity (i.e. lasting longer than three months [6]) can substantially impact daily life and wellbeing [7]. Identifying demographic and socioeconomic characteristics associated with heightened risks of pain worsening and recurrence is therefore important to inform the design of targeted early interventions and address disparities in pain management.

Previous studies examining demographic and socioeconomic differences in pain prognosis generally focus on age, sex and education level [8–24]. The majority of studies investigating long-term pain trajectories use latent trajectory modelling and are consistent in identifying being female as a risk factor for worse long-term pain outcomes; however, results for age and education are mixed [11–15]. Furthermore, while latent trajectory models assume there are several distinct trajectories within a study population, pain trajectories are highly individual. Data sparsity nonetheless usually leads these models to identify mostly linear pain trajectories. As such, they do not fully reflect the dynamic nature of pain conditions over long periods of time, where there may be repeated episodes of worsening and improvement.

In this study, we aimed to understand demographic and socioeconomic differences in the progression and fluctuation of pain over time using 21 years of data from 9369 participants aged ≥50 years in the English Longitudinal Study of Ageing (ELSA). We used multistate models (MSM) to capture the nonlinear and recurrent nature of pain, allowing us to identify groups most at risk of experiencing persistent, worsening, or recurring episodes of pain.

## Methods

### Data sources

ELSA is a nationally representative cohort study of the English population aged ≥50 years. Data collection started in 2002/03 (wave 1) and continued biennially until 2018/19 (wave 9), with wave 10 in 2021/23. Details are available elsewhere [25]. ELSA received ethical approval most recently from the South Central-Berkshire Research Ethics Committee (21/SC/0030). Written informed consent was obtained at each interview. No further ethical approval was required for the present study.

Data from waves 1–10 (2002/03–2021/23) were included in the present study. Participants aged ≥50 years reporting pain for at least one wave with at least two waves of data were eligible for inclusion in analyses.

### Pain assessment

At each interview, participants were asked to report whether they were ‘often troubled with pain’, and how severe the pain was (mild, moderate, severe). For each participant, baseline was defined as the first wave when they reported pain, categorised into moderate–severe or mild pain. At each subsequent wave, participants were again categorised as having moderate-to-severe, mild, or no pain. ELSA does not regularly assess pain duration; both acute and chronic pain were included. Participants were also asked where they experienced pain (all over, back, knee, hip, foot, mouth/tooth, or elsewhere).

### Demographic and socioeconomic factors

We focused on core demographic and socioeconomic factors known to influence pain outcomes in older adults. These factors were assessed at baseline and included age in years, sex (male or female; ELSA survey materials refer to biological sex and not gender), highest educational qualification (low, intermediate, or high, referring to less than secondary, secondary, or above secondary, respectively), marital status (married/partnered or not), and non-pension wealth [26] (referring to the sum of net financial, physical and primary housing wealth excluding pension entitlements; this measure was standardised by year and categorised into quintiles with the first quintile corresponding to the least wealth).

### Statistical methods

**Figure 1:**
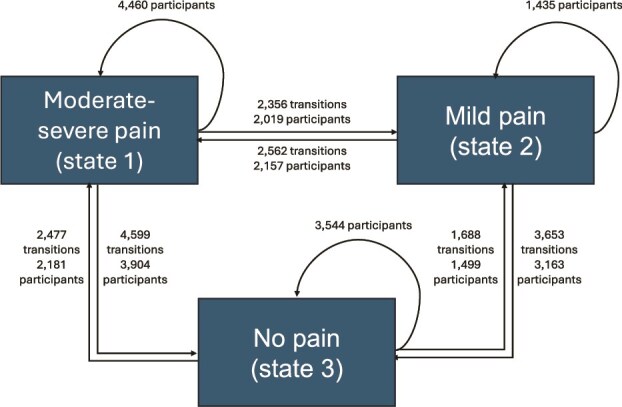
Multistate framework. Arrows indicate observed transitions between states of pain. Numbers on arrows show the total number of transitions and, beneath, the number of unique participants who ever made that transition. Numbers above self-loops indicate participants who remained in the same state.

We used continuous-time MSM [27] to examine associations of demographic and socioeconomic factors with pain transitions. MSMs are used to model the probability of transitioning between predefined states and can handle interval-censoring where the precise time of the state transition is not known (as in ELSA data) and right-censoring [27]. Unlike standard survival models, MSMs allow individuals to contribute multiple transitions between states over time. For the present analysis, we defined three states: (i) moderate–severe pain; (ii) mild pain; and (iii) no pain (Figure 1). Transitions were allowed between all three states.

The follow-up period for MSMs started at first wave of reporting pain for each participant and ended at loss to follow-up or the end of the ELSA study period (May 2023), whichever first occurred. MSMs accommodate intermittent missingness and do not require balanced panel data. If participants missed one or more waves but later re-entered the study, these gaps were treated as interval-censored; if they did not return, follow-up was treated as right-censored at their last observed wave. Individuals with baseline covariate missingness were excluded, given missingness was minor (<5%).

We fitted MSMs with demographic and socioeconomic variables as predictors and extracted hazard ratios (HRs) to examine associations with transitions between states. Four models were used to produce the main results, with the order of including variables intended to account for confounding whilst not adjusting for variables along the causal pathway (Appendix 1, Figure S1): (i) age and sex only; (ii) Model 1 + education; (iii) Model 2 + marital status; and (iv) Model 3 + wealth. Estimates for age and sex were produced using Model 1, education Model 2, marital status Model 3 and wealth Model 4. We did not adjust for health conditions or other factors along the causal pathway from the exposures to pain outcomes as these would obscure effects of interest.

We used models 1–4 to produce HRs for associations of demographic and socioeconomic characteristics with the following transitions: (i) changes in pain severity (worsening pain severity [state 2–1], improving pain severity [state 1–2]); and (ii) pain remission and recurrence (moderate–severe pain remission [state 1–3], moderate–severe pain recurrence [state 3–1]). The MSMs produce HRs for all possible transitions. However, to simplify reporting of the results, we reported moderate–severe (rather than mild) pain remission and recurrence to focus on pain of greater severity more likely to impact wellbeing.

Finally, we used Poisson models including the same covariates as models 1–4 plus adjusted for the logarithm of follow-up duration (to account for differences in follow-up duration) to report associations of demographic and socioeconomic characteristics with number of pain transitions. All analyses were performed in R 4.2.2 with MSMs produced using the *msm* package [27].

### Additional analyses

To examine whether the categorisation of pain severity impacted the results, we first re-ran analyses with pain states re-categorised as: (i) severe pain; (ii) moderate pain; and (iii) no/mild pain [28, 29].

Second, we aimed to better approximate chronic pain by rerunning analyses including only participants who reported pain at baseline and continued to report pain at the subsequent wave (i.e. reported pain for at least two consecutive waves).

## Results

### Participant characteristics

Of 11 912 ELSA respondents aged ≥50 and reporting pain, 417 (3.5%) were missing demographic or socioeconomic characteristics, and 2126 (17.8%) had one wave of data and were excluded, leading to 9369 participants aged 50–98 at baseline included in analyses. Excluded ELSA respondents were mostly similar with respect to demographic and socioeconomic characteristics to those included (Appendix 1, Table S1).

Participant characteristics at baseline are shown in Table 1. At baseline, 5872 (62.7%) of 9369 participants reported moderate–severe and 3497 (37.3%) reported mild pain. The moderate–severe pain group was older than the mild pain group and more likely to be female, single, less educated and less wealthy (*P* < .0001 for all). In total, 76.9% of participants reported back, hip, knee, or foot pain (Appendix 1, Table S2).

**Table 1:** Participant characteristics at baseline (*N* = 9369)

	**Moderate–severe pain** (*N* = 5872)	**Mild pain** (*N* = 3497)	*P-value*
Age in years, mean (SD)	64.2 (9.9)	62.9 (9.4)	<.0001
Sex			
Male	2311 (39.4)	1680 (48.0)	<.0001
Female	3561 (60.6)	1817 (52.0)	
Marital status			
Single	1773 (30.2)	772 (22.1)	<.0001
Married/partnered	4099 (69.8)	2725 (77.9)	
Highest educational qualifications			
Less than secondary (low)	2828 (48.2)	1125 (32.2)	
Secondary (intermediate)	2447 (41.7)	1701 (48.6)	<.0001
Above secondary (high)	597 (10.2)	671 (19.2)	
Wealth quintile			
1 (Lowest)	1240 (21.1)	443 (12.7)	<.0001
2	1213 (20.7)	573 (16.4)	
3	1238 (21.1)	675 (19.3)	
4	1162 (19.8)	829 (23.7)	
5 (Highest)	1019 (17.4)	977 (27.9)	

N (%) unless otherwise indicated. Baseline refers to first wave at reporting pain for each participant.

Abbreviation: SD, standard deviation.

Follow-up duration was similar for both pain intensity groups (median = 9.4 years, interquartile range [IQR] = 4.3–13.8 in the moderate–severe pain group; median = 9.6, IQR = 5.2–13.8 in the mild pain group; Appendix 1, Table S3).

### Transitions between pain states

Over up to 21 years of follow-up during which participants were observed 49,273 times, 17,335 transitions between states occurred (Figure 1; Appendix 1, Table S4). In total, 2562 (14.8%) of these transitions were from mild to moderate–severe pain and 2356 (13.6%) were from moderate–severe to mild pain. There were also 4599 (26.5%) transitions from moderate–severe pain to no pain (‘remission’) and 2477 (14.3%) transitions from no pain back to moderate–severe pain (‘recurrence’).

During the study period, 3116 (33.3%) of 9369 participants had one transition between states, 1740 (18.6%) two, 1297 (13.8%) three, 742 (7.9%) four and 703 (7.5%) five or more transitions; 1771 (18.9%) participants remained in the same state for the entire study period. In Poisson models, older age, more education and higher wealth were associated with making more transitions, while female sex was associated with making fewer transitions; marital status was not associated with the number of transitions made (Appendix 1, Table S5).

#### Changes in pain severity

**Figure 2:**
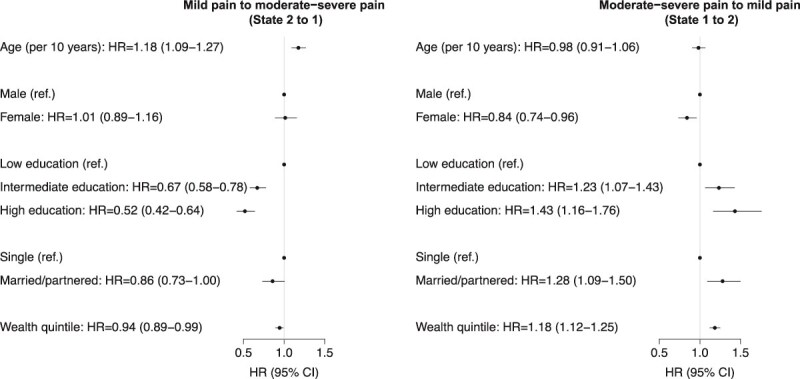
Hazard ratios for changes in pain severity (*N* = 9369). ‘Low’ education refers to less than secondary education, ‘intermediate’ secondary education and ‘high’ above secondary education. Hazard ratio for wealth corresponds to a quintile increase in wealth. Abbreviations: HR, hazard ratio; CI, confidence interval.

HRs for worsening pain severity (mild to moderate–severe pain) are shown in Figure 2 (left panel). Participants who were older at baseline were more likely to have worsening pain severity (HR for 10-year increase in age = 1.18, 1.09–1.27). Higher levels of education were associated with lower risk of pain worsening (with low education as the reference, HR for intermediate education = 0.67, 0.58–0.78; or 0.52, 0.42–0.64 for high education). Finally, wealthier participants were less likely to have worsening pain (HR for quintile increase in wealth = 0.94, 0.89–0.99).

HRs for improvements in pain severity (moderate–severe to mild pain) are shown in Figure 2 (right panel). Females were less likely than males to experience pain improvements (HR = 0.84, 0.74–0.96). Compared to those with low education, individuals with intermediate (HR = 1.23, 1.07–1.43) and high (HR = 1.43, 1.16–1.76) education were both more likely to have improvements in pain severity. Married/partnered participants were more likely than single participants to experience pain improvements (HR = 1.28, 1.09–1.50). Finally, wealthier participants were more likely to experience pain improvements (HR = 1.18, 1.12–1.25).

There were no other associations of demographic and socioeconomic characteristics with changes in pain severity (Figure 2).

#### Pain remission and recurrence

**Figure 3:**
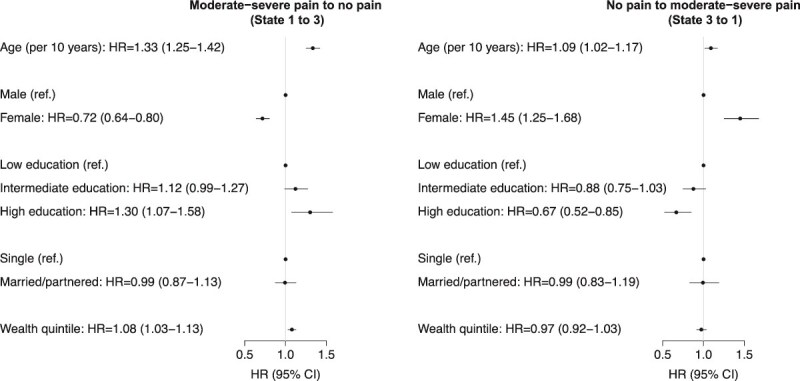
Hazard ratios for moderate-to-severe pain remission and recurrence (*N* = 9369). ‘Low’ education refers to less than secondary education, ‘intermediate’ secondary education and ‘high’ above secondary education. Hazard ratio for wealth corresponds to a quintile increase in wealth. Abbreviations: HR, hazard ratio; CI, confidence interval.

HRs for pain remission (moderate–severe pain to no pain) are shown in Figure 3 (left panel). Individuals older at baseline were more likely to experience remission (HR = 1.33, 1.25–1.42). Females were less likely than males to experience remission (HR = 0.72, 0.64–0.80). More educated participants were more likely to experience remission (HR intermediate education = 1.12, 0.99–1.27; HR high education = 1.30, 1.07–1.58). Wealthier participants were also more likely to experience remission (HR = 1.08, 1.03–1.13).

HRs for pain recurrence (no pain to moderate–severe pain) are shown in Figure 3 (right panel). Higher age at baseline was associated with increased risk of recurrence (HR = 1.09, 1.02–1.17). Females were more likely than males to have pain recurrence (HR = 1.45, 1.25–1.68). Finally, more educated participants were less likely to experience pain recurrence (HR for intermediate education = 0.88, 0.75–1.03; HR high = 0.67, 0.52–0.85).

There were no other associations of demographic and socioeconomic factors with pain remission or recurrence (Figure 3).

### Additional analyses

Conclusions were unchanged when pain states were categorised as severe pain, moderate pain and mild/no pain (Appendix 1, Figure S2) and when analyses were restricted to participants with suspected chronic pain (Appendix 1, Figure S3).

## Discussion

In this multistate analysis of changes in pain severity and recurrence over two decades, two key findings emerged. First, relatively few individuals remained in a stable pain state, with just under half of participants experiencing two or more transitions during follow-up. Second, sex and socioeconomic indicators showed the most consistent associations with pain transitions. Women and individuals with less education or wealth had more adverse long-term pain trajectories—characterised by less improvement or remission and more frequent worsening or recurrence. In contrast, pain outcomes were generally more favourable among men and those with greater socioeconomic advantage. Taken together, the results suggest that while long-term pain trajectories fluctuate considerably for everyone, these fluctuations more often involve worsening, persistent, or recurring pain among women and individuals in socioeconomically disadvantaged positions.

In accordance with the present study, previous studies have consistently demonstrated that women with pain are less likely to recover [10, 16, 18, 20] and more likely to have pain recurrence [19] compared with men. These disparities may in part reflect structural barriers and healthcare inequities—for example, women are less likely to be referred for joint replacement despite greater need [30]—and provider bias, including minimisation of pain [31]. Biological factors may also contribute, including increased susceptibility to poorly understood conditions like fibromyalgia [31], and sex-specific conditions such as perimenopausal pain and endometriosis [32, 33]. Together, these issues could translate to less effective pain management [34].

Findings are mixed for other demographic and socioeconomic factors, and most studies report results for age and education level only. Some studies find older adults have slower [10, 17, 18] or faster [15, 16] recovery from musculoskeletal pain, while other studies find no age association with pain severity trajectory [14], remission [8, 9], or spread [8, 12, 13]. Differences in findings are likely due to varying definitions of pain, follow-up periods, and differences in the included age range. In our study of general pain in adults aged 50–98 years at baseline followed up over up to 21 years, we found that increasing age was associated with pain worsening, remission and recurrence. This suggests pain might fluctuate more in older adults, which could reflect the additional challenges of effective pain management with ageing [35]. Nevertheless, the results for age could also reflect survival bias, where older adults who enrol in ELSA might be somewhat healthier than average and therefore more likely to experience pain remission. Mortality could have also affected results for age; we could not include death as an absorbing state due to incomplete mortality data during the study period.

Previous studies examining education also give inconsistent results. One study suggests that less education is associated with worse general pain severity trajectories [11], whereas other studies find no association with pain recovery or recurrence [8, 10]. In the present study, we found that individuals with higher levels of education had lower risks of pain worsening and recurrence, and were more likely to improve and to experience remission. We found a similar trend for wealth. Socioeconomic advantage is associated with better health literacy [36], increased access to supplementary treatments and therapies [37], and better social support networks [38, 39], all of which might facilitate better pain management. The results for marital status—where married/partnered participants were more likely to have improving pain—suggest a role of social support in long-term pain trajectories. Finally, pain severity is inherently subjective. Accordingly, differences in trajectories may reflect changes in underlying pathology and nociceptive input, as well as differences in pain processing such as sensitivity and tolerance.

The main strength of our study is its use of MSM and data drawn from a largescale nationally representative study with a follow-up period of two decades. Using MSM allowed us to model highly variable pain trajectories, which are inadequately captured in previous work. Our use of data from a nationally representative cohort study increases the generalisability of the results relative to previous studies in clinical populations. The long follow-up period allowed us to examine multiple transitions between states of pain severity. We were able to focus on an age range where pain is highly prevalent, and identifying predictors of pain progression is therefore of particular relevance for policy and practice.

There are also several limitations. As noted above, our use of data from a nationally representative cohort increases generalisability relative to clinical studies. However, within ELSA, participants were required to contribute at least two waves of data to be included in the analysis. Although the analytic sample was similar to the full ELSA cohort with respect to demographic and socioeconomic characteristics, health status is another important driver of attrition in ageing cohorts [40, 41], and demographic comparability does not guarantee comparable health. The analytic sample may therefore still be healthier than the full ELSA cohort, which could reduce generalisability to the general population. Due to data limitations, we could not distinguish between chronic and other pain; however, our sensitivity analysis suggested results were relevant for chronic pain. We could not consider race or ethnicity because ELSA is 95% white, reflecting the demographic characteristics of adults aged 50 years and older in England during the study period [42]. Future studies should examine the generalisability of the results in more diverse study populations, further clarify differences between acute and chronic pain, how results differ for different types of pain, and the role of pain treatments, therapies and interventions such as joint replacement, which we could not comprehensively examine with the available data.

The findings of this study highlight the importance of considering demographic and socioeconomic factors in pain management, particularly given the highly variable nature of pain trajectories. Policies should focus on improving access to pain management resources for individuals of lower socioeconomic position and consider the intersection of age, sex/gender and socioeconomic position, where compounding disadvantage might exacerbate inequalities. Gender-sensitive approaches are also necessary to address disparities in long-term pain, ensuring that pain in women is neither underestimated nor undertreated. These could include adapting diagnostic criteria to account for sex-specific symptoms and pain responses, and educating health care practitioners to recognise gender biases that contribute to health disparities [43]. Finally, given the apparent protective role of social support, integrating social prescribing initiatives—where patients are connected with peer support groups, caregiver networks, or community resources—could be evaluated for its potential to improve long-term pain outcomes.

Taken together, the findings of this study demonstrate that socioeconomic and sex disparities in pain extend far beyond onset to shape the entire trajectory of pain. Distinguishing between onset and subsequent trajectories is key: if disadvantaged groups are systematically less likely to recover and more likely to relapse, pain care that focuses only on prevention of onset will be insufficient. Health systems must incorporate proactive, equity-informed follow-up to prevent pain inequities from compounding over time.

### Research data transparency and availability

ELSA data are available to researchers after registration with the UK data service at https://beta.ukdataservice.ac.uk/datacatalogue/series/series?id=200011.

## Supplementary Material

aa-25-1817-File005_afaf364
