# Supplementary material for: Demographic and socioeconomic risk factors for pain progression and recurrence in middle-aged and older adults: multistate analysis of a prospective English cohort study
Source: Age Ageing. 2026 Jan 3;55(1):afaf364. doi: 10.1093/ageing/afaf364 (PMC12763816; doi:10.1093/ageing/afaf364)
Supplement: aa-25-1817-File005_afaf364 [file aa-25-1817-file005_afaf364.pdf]

Demographic and socioeconomic factors associated with chronic pain progression and recurrence in middle-aged and older adults: multistate analysis of a prospective English cohort study

**Appendix 1. Supplementary materials.**

*Contents*

Supplementary figures. ........ 2

Figure S1. Directed acyclic graph. ........ 2

Figure S2. Hazard ratios for transitions with state 1: severe pain; state 2: moderate pain; state 3: mild/no pain (N=9,369). ........ 3

Figure S3. Hazard ratios for transitions when analysis is restricted to longer-term pain (N=5,357). ........ 3

Supplementary tables. ........ 5

Table S1. Characteristics of ELSA participants at baseline included and excluded from analysis. ........ 5

Table S2. Location of pain at baseline. ........ 6

Table S3. Waves of data per participant. ........ 7

Table S4. Number of transitions between states in the analytic sample. ........ 8

Table S5. Associations of demographic and socioeconomic characteristics with number of transitions made during the follow-up period. ........ 9

**Supplementary figures.**

Figure S1. Directed acyclic graph.

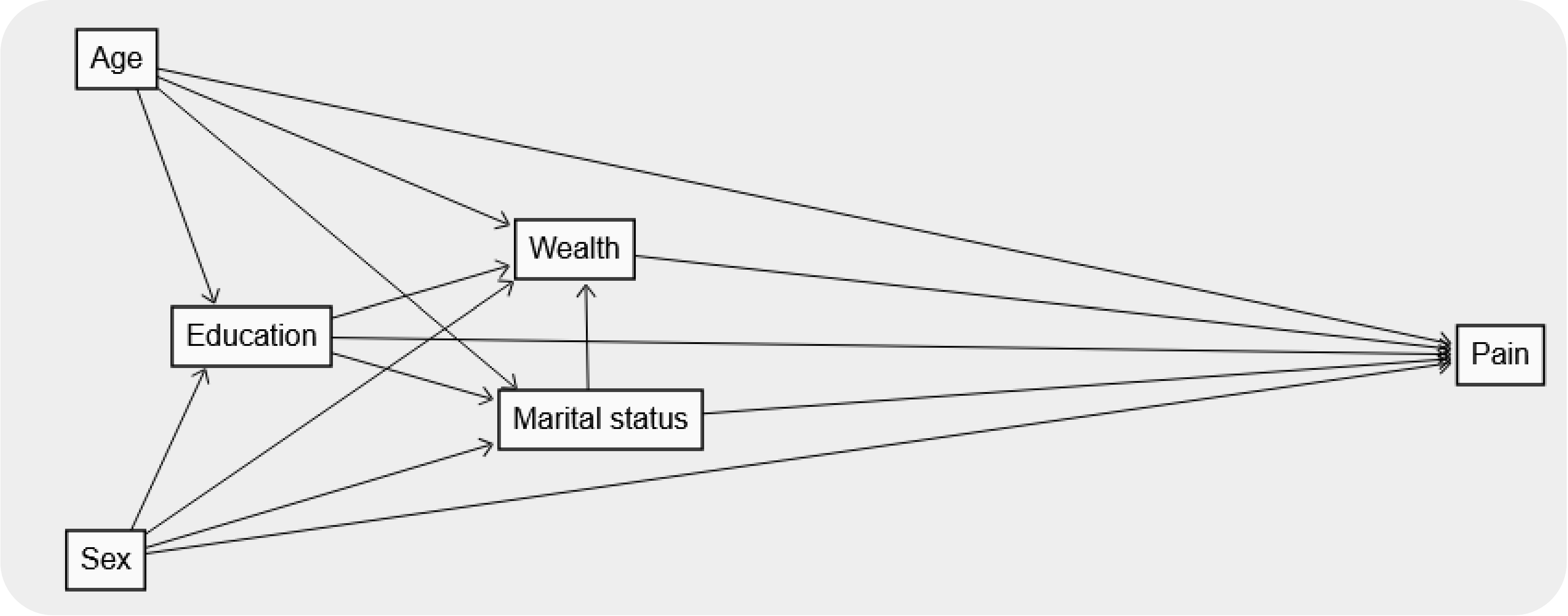

Figure S2. Hazard ratios for transitions with state 1: severe pain; state 2: moderate pain; state 3: mild/no pain (N=9,369).

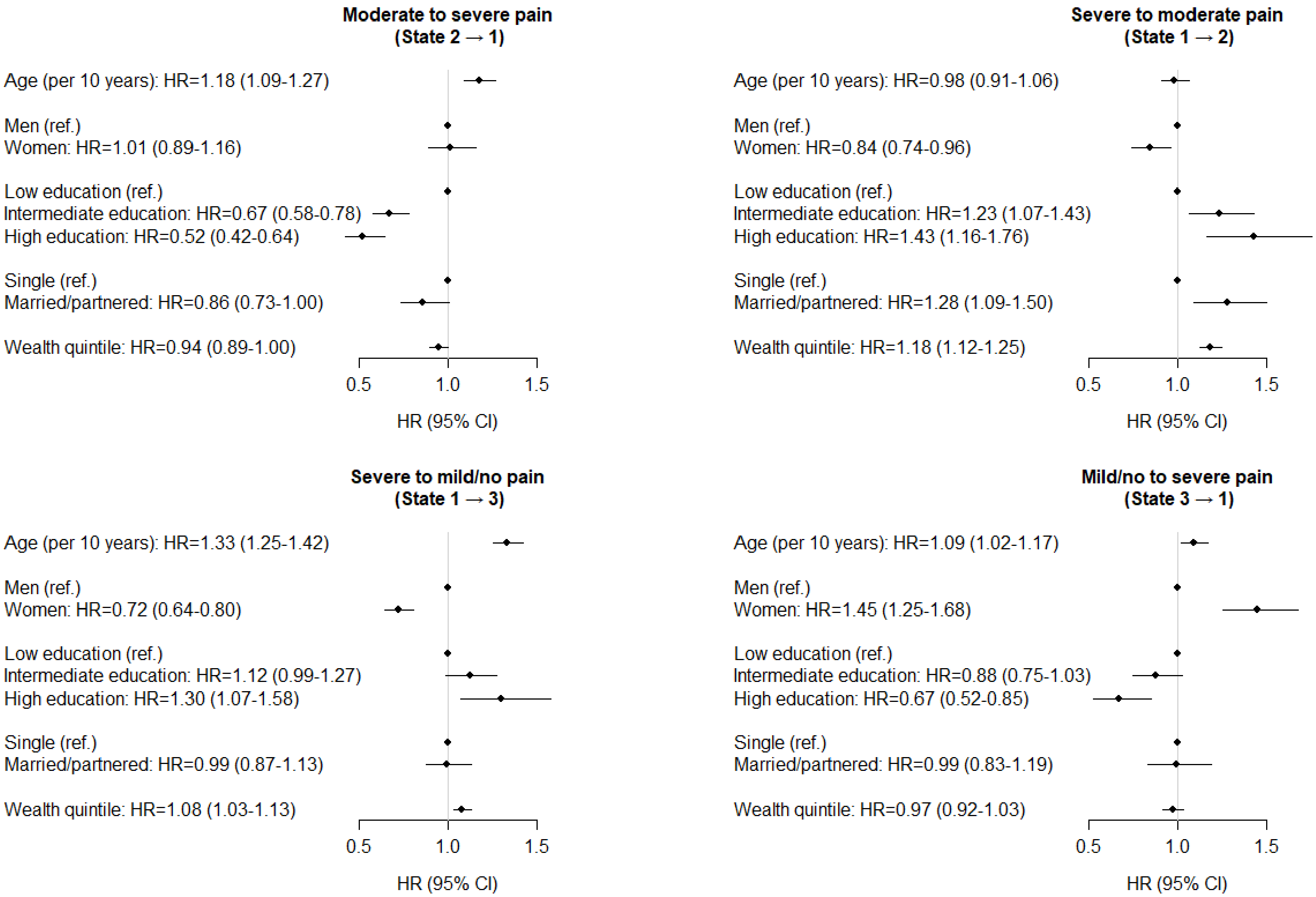


'Low' education refers to less than secondary education, 'intermediate' secondary education, and 'high' above secondary education. Hazard ratio for wealth corresponds to a quintile increase in wealth. Abbreviations: HR, hazard ratio; CI, confidence interval.

Figure S3. Hazard ratios for transitions when analysis is restricted to longer-term pain (N=5,357).

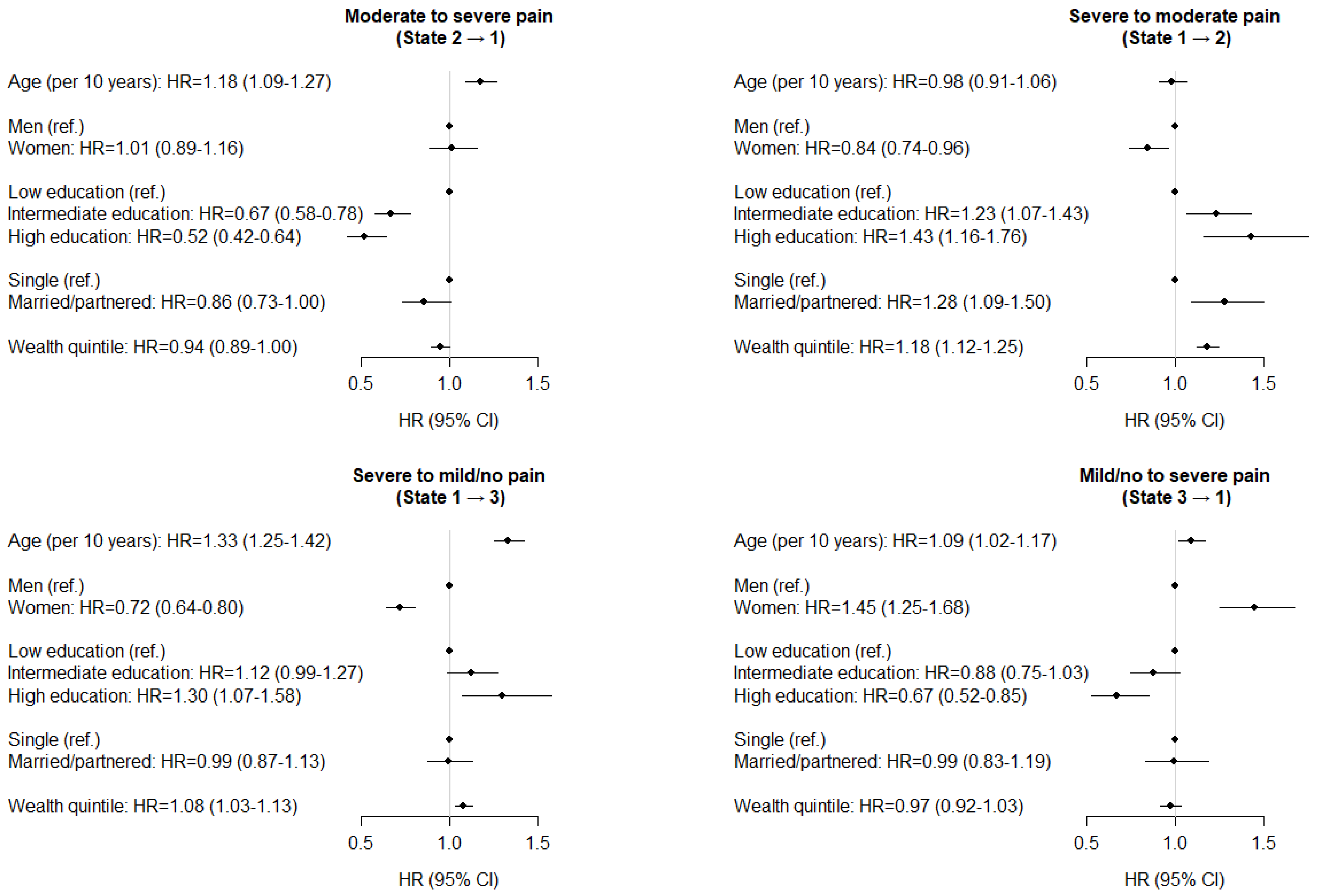


'Low' education refers to less than secondary education, 'intermediate' secondary education, and 'high' above secondary education. Hazard ratio for wealth corresponds to a quintile increase in wealth. Abbreviations: HR, hazard ratio; CI, confidence interval.

**Supplementary tables.**

Table S1. Characteristics of ELSA participants at baseline included and excluded from analysis.

| | **Included** (N=5,872) | **Excluded** (N=3,497) |
|---|---|---|
| Age in years, mean (SD) | 63.7 (9.7) | 65.4 (11.6) |
| Sex | | |
| Male | 3991 (42.6) | 1166 (45.8) |
| Female | 5378 (57.4) | 1375 (54.0) |
| Missing | 0 (0.0) | 3 (0.1) |
| Marital status | | |
| Single | 2545 (27.2) | 825 (32.4) |
| Married/partnered | 6824 (72.8) | 1716 (67.5) |
| Missing | 0 (0.0) | 3 (0.1) |
| Highest educational qualifications | | |
| Less than secondary | 3953 (42.2) | 1156 (45.4) |
| Secondary | 4148 (44.3) | 1063 (41.8) |
| Above secondary | 1268 (13.5) | 293 (11.5) |
| Missing | 0 (0.0) | 32 (1.3) |
| Wealth quintile | | |
| 1 (Lowest) | 1683 (18.0) | 668 (26.3) |
| 2 | 1786 (19.1) | 561 (22.1) |
| 3 | 1913 (20.4) | 435 (17.1) |
| 4 | 1991 (21.3) | 356 (14.0) |
| 5 (Highest) | 1996 (21.3) | 356 (14.0) |
| Missing | 0 (0.0) | 168 (6.6) |
| Pain level | | |
| None | 0 (0.0) | 3 (0.1) |
| Mild pain | 3497 (37.3) | 853 (33.5) |
| Moderate-severe pain | 4314 (46.0) | 1162 (45.7) |

N (%) unless otherwise indicated.
Abbreviations: SD, standard deviation.

Table S2. Location of pain at baseline.

| Pain location | N | Percent |
|---|---|---|
| All over | 183 | 2.0 |
| Back/hip/knee/feet | 7,204 | 76.9 |
| Mouth/tooth | 33 | 0.4 |
| Elsewhere | 1,949 | 20.8 |

Table S3. Waves of data per participant.

| Number of waves | N | Percent |
|---|---|---|
| 2 | 1,604 | 17.1 |
| 3 | 1,316 | 14.1 |
| 4 | 1,182 | 12.6 |
| 5 | 1,138 | 12.2 |
| 6 | 1,069 | 11.4 |
| 7 | 1,022 | 10.9 |
| 8 | 789 | 8.4 |
| 9 | 671 | 7.2 |
| 10 | 578 | 6.2 |

Table S4. Number of transitions between states in the analytic sample.

| From \ To | State 1: Moderate-severe pain | State 2: Mild pain | State 3: No pain | Total |
|---|---|---|---|---|
| State 1: Moderate-severe pain | 11948 (63.2) | 2356 (12.5) | 4599 (24.3) | 18903 (100) |
| State 2: Mild pain | 2562 (30.5) | 2191 (26.0) | 3653 (43.5) | 8406 (100) |
| State 3: No pain | 2477 (19.7) | 1688 (13.4) | 8430 (66.9) | 12595 (100) |

N (row percentage) shown.

Table S5. Associations of demographic and socioeconomic characteristics with number of transitions made during the follow-up period.

| | IRR (95% CI) | P-value |
|---|---|---|
| **Model 1** | | |
| *Age (per 10 years)* | 1.04 (1.02-1.06) | <0.0001 |
| *Sex* | | |
| Male | Ref. | |
| Female | 0.93 (0.91-0.96) | <0.0001 |
| **Model 2** | | |
| *Education* | | |
| Less than secondary | Ref. | |
| Secondary | 1.06 (1.03-1.10) | 0.00026 |
| Above secondary | 1.08 (1.04-1.14) | 0.00065 |
| **Model 3** | | |
| *Marital status* | | |
| Single | Ref. | |
| Married/partnered | 1.01 (0.98-1.06) | 0.29 |
| **Model 4** | | |
| *Wealth quintile* | 1.03 (1.02-1.05) | <0.0001 |

Based on Poisson models adjusted for the logarithm of follow-up duration and all terms in previous model (e.g., Model 2 is adjusted for Model 1 terms).
Abbreviations: IRR, incidence rate ratio; CI, confidence interval.
